# New Pharmacological Perspectives for the Leptin Receptor in the Treatment of Obesity

**DOI:** 10.3389/fendo.2014.00167

**Published:** 2014-10-13

**Authors:** Clara Roujeau, Ralf Jockers, Julie Dam

**Affiliations:** ^1^INSERM, U1016, Institut Cochin, Paris, France; ^2^CNRS UMR 8104, Paris, France; ^3^University of Paris Descartes, Sorbonne Paris Cité, Paris, France

**Keywords:** leptin, obesity, leptin receptor, diabetes, leptin resistance

## Abstract

After its discovery in 1994, leptin became the great hope as an anti-obesity treatment based on its ability to reduce food intake and increase energy expenditure. However, treating obese people with exogenous leptin was unsuccessful in most cases since most of them present already high circulating leptin levels to which they do not respond anymore defining the so-called state of “leptin resistance.” Indeed, leptin therapy is unsuccessful to lower body weight in commonly obese people but effective in people with rare single gene mutations of the leptin gene. Consequently, treatment of obese people with leptin was given less attention and the focus of obesity research shifted toward the prevention and reversal of the state of leptin resistance. Many of these new promising approaches aim to restore or sensitize the impaired function of the leptin receptor by pharmacological means. The current review will focus on the different emerging therapeutic strategies in obesity research that are related to leptin and its receptor.

## Introduction

Obesity being one of the major issues of public health nowadays, with more than 500 million obese adults worldwide in 2011, a lot of effort has been made to elaborate anti-obesity therapies. Obesity is a chronic disease, which originates from a myriad of causes leading to a steady imbalance between energy intake and energy expenditure. The resulting excess of fat mass has been correlated to higher risk of developing severe diseases such as type 2 diabetes, cardiovascular disease, and cancers. There is currently no successful long-term treatment against obesity with the exception of bariatric surgery, which is costly with potential risks and complications. Obesity being a complex disease originating from multiple environmental and genetic factors, an efficient pharmacological cure against obesity is still not available. A pharmacological drug against obesity must result in a significant reduction of food intake and/or an increase in energy expenditure whatever the mechanistic target of the drug (1). Given that dietary intervention result often in no or very modest long-lasting weight loss and that previous marketed drugs showed poor efficacy with high risk of side effects, innovative therapies with high efficacy, and maximal safety are urgently needed. The discovery of leptin in 1994 raised the possibility of new therapeutic strategies to combat obesity epidemic (2). Administration of recombinant leptin to obese rodents and humans with congenital leptin deficiency significantly decreases body weight and food intake (3–5). Moreover, leptin therapy for patients with very low leptin or leptin deficiency has proven to be relevant for diseases such as lipoatrophy, anorexia nervosa, hypothalamic amenorrhea, and some neuroendocrine disturbances (6). However, recombinant leptin monotherapy is totally inefficient in decreasing body weight of diet-induced obese (DIO) mice as well as obese humans who are not leptin-deficient but rather hyperleptinemic, associated with a loss of responsiveness to leptin (7). These observations led to the concept of so-called “leptin resistance.” The prevention and reversal of leptin resistance represents a major challenge in obesity research. Understanding the biological function of leptin and its receptor (OBR) is an important step in order to conceive efficient and specific therapeutic tools that would rescue the impaired function of the leptin system observed in obesity. Persistent efforts have been pursued both at the level of deciphering the biology of leptin and in designing therapeutic solutions. This review summarizes recent findings on OBR functions and possible directions for therapeutic perspectives.

## Few Words on Leptin and the Leptin Receptor

Leptin, a peptidic hormone mainly secreted by white adipose tissue, is essential in the control of energy homeostasis (8). The central regulation of food intake and energy expenditure is mediated through the binding of leptin to its receptor OBR, a type I cytokine receptor. Several isoforms of OBR have been described as a result of alternative mRNA splicing leading to several short isoforms (OBRa, OBRc, OBRd, and OBRf), one long isoform OBRb with a long cytosolic C-terminus tail and one soluble isoform OBRe (9). Hence, OBR isoforms differ in the length of their intracellular region but share identical extracellular domains. While short isoforms are ubiquitously expressed, OBRb expression is more restricted with high levels in hypothalamic nuclei such as the arcuate nucleus (ARC). The hypothalamic ARC has an important role in the development of leptin resistance. Accordingly, exposure of rodents to a high-fat diet rapidly decreases the phosphorylation of STAT3 in the ARC or the ventral tegmental area (VTA), while leptin-sensitivity is simultaneously maintained in some other hypothalamic nuclei (10, 11). While the biological function of the short isoforms is still elusive, it is well established that OBRb is the main isoform responsible for the effect of leptin on body weight control (12). The weight lowering properties of leptin via OBRb has been suggested to be centrally mediated (13–15). Once activated after leptin binding, OBRb is able to trigger various signal transduction pathways. Activation of the Janus tyrosine kinase 2 (JAK2)/signal transducer and activator of transcription 3 (STAT3) pathway leads to an increase of anorexigenic signals and a decrease of orexigenic signals (16). Leptin is also able to activate the insulin receptor substrate (IRS)/phosphatidylinositide 3-kinase (PI3K) pathway, essential for the regulation of glucose homeostasis (17). Moreover, leptin inhibits in the brain the energy sensor, adenosine monophosphate-activated protein kinase (AMPK), to decrease eating (18, 19). The activation of extracellular signal-regulated kinase (ERK) is another pathway mediating the anorectic action of leptin in the hypothalamus (20).

**Figure 1:**
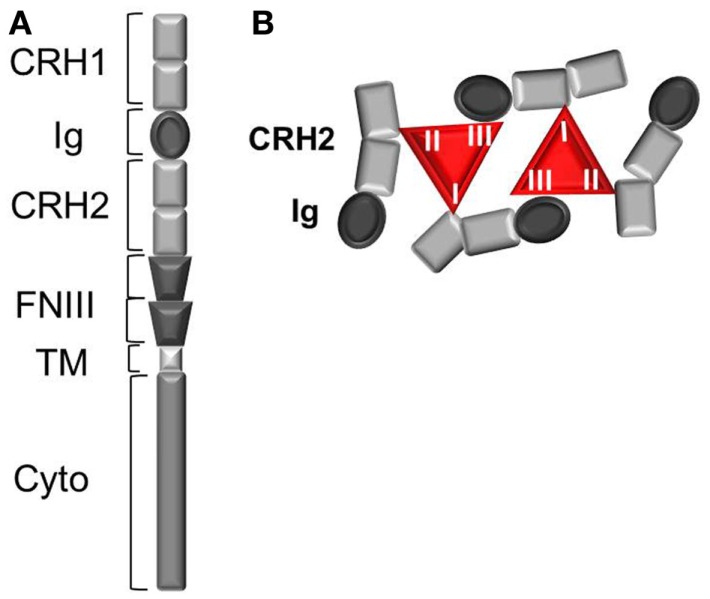
**The leptin receptor (OBR)**. **(A)** Schematic view of OBR protomer. CRH, cytokine receptor homology domain; Ig, immunoglobulin domain; TM, transmembrane region; FNIII, fibronectin III domain. **(B)** Leptin:OBR hexameric complex based on biochemical and molecular modeling data. In the hexameric complex, two molecules of leptin interact with four OBR protomers, where each leptin molecule binds OBR through the high affinity binding site II (to CRH2) and via the two lower affinity binding sites I (to CRH2) and III (to Ig domain).

Leptin binding to its receptor OBR is the first event triggering conformational change and eventual oligomerization of OBR, both necessary for receptor activation and subsequent signal transduction. Understanding the mechanism of OBR activation and signaling is an essential step in order to better apprehend the causes leading to an impairment of OBR function in human obesity. OBR exists as a pre-formed dimer (or oligomer) that was suggested to be disulfide-linked (21). OBR N-terminal region is composed of one conserved cytokine receptor homologous domain (CRH1), a conserved immunoglobulin (Ig) domain, followed by another cytokine receptor homology domain (CRH2) and two fibronectin type 3 (FNIII) domains, which are located proximal to the transmembrane fragment (Figure 1). The CRH2 and Ig domains are involved in leptin binding and are required for receptor activation. A combination of ligand binding studies, site directed mutagenesis and homology molecular modeling suggested the existence of three different binding sites (I, II, and III) on the leptin molecule, similar to the interleukin 6-receptor family. Binding site II is indispensable for high affinity interaction (in the nanomolar range) between leptin and the OBR CRH2 domain (22, 23). Binding site III is involved in the conformational changes and activation of OBR by interacting with the Ig domain. The role of binding site I is still poorly defined. Modified leptin with an intact site II but with mutations in site I or site III still binds the CRH2 domain with strong affinity but lost the capacity to activate OBR (24–26). Hence, leptin mutations at site I (L39A/D40A/F41A) and III (S120A/T121A) become antagonists (23–25). In accordance with this, leptin mutants on binding site I increase body weight gain in rodents as expected by competing with endogenous leptin (27). According to the current model of OBR activation, binding of two leptin molecules to OBR extracellular domains are believed to trigger a conformational change and dimer clustering into tetramers to form an hexameric complex with two leptin molecules for four receptor protomers (Figure 1) (28, 29). Recently, the architecture of the extracellular region of OBR, alone and in complex with leptin, characterized by single-particle electron microscopy, challenged this view (30). Electron microscopy results showed that leptin binding site III, as foreseen in the activation model, serves to recruit the Ig domain of a second receptor chain. The data also suggested that leptin and OBR interact in a tetrameric complex, with a 2:2 stoichiometry, by engaging only leptin sites II and III, excluding the existence of binding site I. The role of binding site I in complex formation and the way it is involved in the activation mechanism still remain elusive. Lately, Small Angle X-ray Scattering (SAXS) provided structural insight into the activated architecture of OBR. The two well-known non-functional leptin antagonists (L39A/D40A/F41A) or (S120A/T121A) interact with OBR with a stoichiometry of 1:1 in contrast to wild type leptin, confirming that those two sites are required for the formation of a higher order clustering necessary for OBR activation (4:4 stoichiometry) (31). In addition to the induction of receptor clustering by leptin through binding to CHR2, leptin leads to the rigidification of the flexible hinge of CHR2 and favors a certain orientation of the FNIII domains that will be transmitted to the transmembrane segments (30). The importance of adequate positioning of FNIII domains is highlighted by the fact that OBR activation can be inactivated by anti-OBR antibodies directed against the FNIII domains (26). Knowing the structure of OBR is an important prerequisite for a better understanding of the functional modifications that may occur at the receptor level during the process of leptin resistance impairing OBR function.

**Figure 2:**
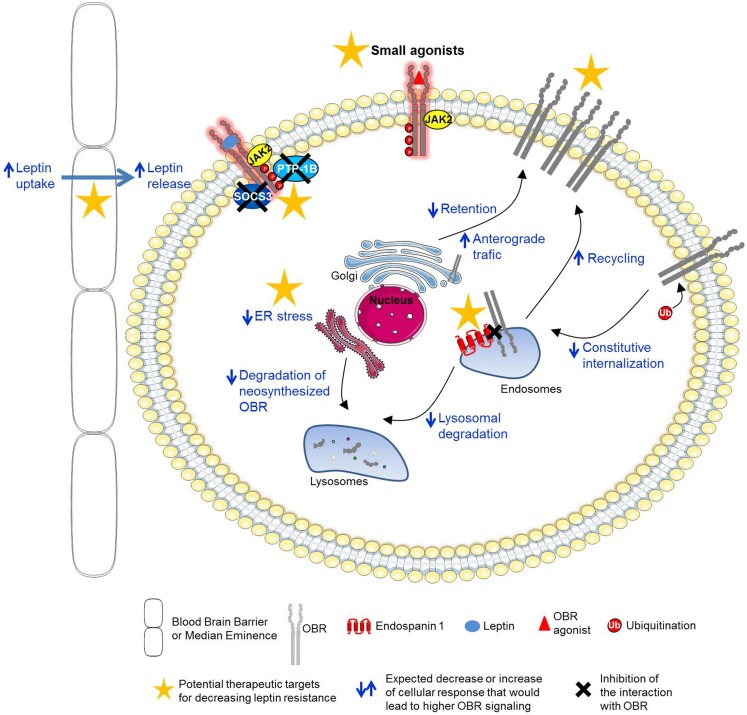
**Potential therapeutic targets for the prevention or reversal of leptin resistance**. Potential therapeutic strategies to prevent or reverse leptin resistance: (1) increase of leptin signaling with new OBR agonists, (2) decrease of the inhibitory function of OBR negative regulators (SOCS3, PTP1B), (3) increase of leptin transport across the blood–brain barrier or median eminence, (4) increase of OBR cell surface expression by enhancing OBR anterograde traffic and recycling or by decreasing OBR constitutive internalization and lysosomal degradation (endospanin 1-dependent pathways), and (5) decrease of ER stress.

Single genes whose mutations lead to “monogenic” forms of obesity are very rare. Among them, mutations in the leptin gene (*ob/ob* mice) or in the leptin receptor gene (*db/db* mice, *fa/fa* rats), leading to the deficiency of the related proteins, produce extreme obesity in rodents (9, 32–35) as well as in humans (36–39), due to hyperphagia, decreased energy expenditure, and insulin-resistance. Naturally occurring mutations (A409E and R612H) on human OBR lead to severe obesity in patients possibly by impairing binding site III and binding site II, respectively (40). However, genetic alterations are rare and paradoxically most obese humans have high levels of plasma leptin, proportional to the excess of fat mass, and are less responsive to the action of leptin, due to a decreased sensitivity to the hormone. Clearly dietary macronutrients participate in the development of leptin resistance, especially fat and sugar are detrimental to leptin sensitivity (41–44). The molecular basis for this leptin resistance is not yet completely understood but considerable efforts have been made in this direction. Several studies led to the hypotheses that leptin resistance could arise from an alteration of multiple mechanisms, such as a decreased transport of leptin into the brain (45–50), an impairment of neuronal plasticity (51–53), an over-activation of inhibitory signals of leptin signaling (54–58), hyperleptinemia (59), a defect in OBR trafficking (60–62), and endoplasmic reticulum (ER) stress (63–66) (Figure 2). A better understanding of those mechanisms prompted researchers to elaborate new therapeutic strategies to reverse or prevent leptin resistance. Design of potential therapeutic solutions is discussed below.

## Leptin Sensitizing Molecules

Because of its crucial role in controlling satiety and body weight, leptin triggered a frenetic enthusiasm, after its discovery in 1994, with the perspective of using leptin therapy to treat obesity. Few years later, Farooqi and colleagues found that daily subcutaneous injections of recombinant human leptin led to decreased hyperphagia and weight loss in severely obese children carrying mutations in the leptin gene (67, 68). Leptin administration has also been proven effective in animals and patients with lipodystrophy, a disease characterized by almost absence of fat mass and therefore low secretion of leptin resulting in excessive calories intake, which are stored as fat in liver and muscle leading to type II diabetes and high blood lipid levels (69, 70), or for the treatment of anorexia nervosa (71, 72). However, hope was rapidly lost in the late 1990s after the failure of leptin to induce significant weight loss in most overweight and obese people, who are typically leptin-resistant (73). Recently, leptin replacement showed promising results when associated with reduced caloric intake. Indeed, appearance of metabolic responses seen as a metabolic adaptation, defending against changes of energy status during diet intervention, leads to a resistance to weight loss (74, 75). The drop of plasma leptin occurring with prolonged energy deficit during weight loss is part of this metabolic adaptation (76–78). Hence, leptin replacement during weight loss associated with a decrease in plasma leptin is able to further reduce and maintain weight loss in rodents (79, 80) and humans (75, 81). However, those effects are only minimal and it appears that leptin replacement is not sufficient by itself. Several studies turned toward combinatorial therapies in which the targeting of more than one hormonal pathway should show better efficacy. Indeed, the simultaneous targeting of several systems with multi-therapy can (i) target several mechanisms of action at the same time and (ii) minimize the establishment of potential compensatory mechanisms. Hence, a combination of leptin with molecules capable of improving or restoring central leptin-sensitivity, constitutes an extremely promising pharmacological treatment for body weight loss. Peptide hormones that are not toxic and have known physiological roles in the regulation body weight are of particular interest.

Several studies analyzed the effects of a combination of leptin with amylin, a 37-amino acid anorexigenic hormone, co-secreted with insulin from β-pancreatic cells in response to food consumption. Amylin receptors are widespread in the brain and regulate energy balance and glucose homeostasis (82). Amylin receptors consist of heterodimerized complexes of the calcitonin receptor (CTR), a seven transmembrane-domain G protein-coupled receptor, which interacts with receptor activity-modifying proteins (RAMPs) (83, 84). Studies from Amylin Pharmaceuticals, have shown that in leptin-resistant, high-fat DIO rats, a combination of amylin and leptin therapy results in greater inhibition of food intake and body weight loss, compared to monotherapy conditions, suggesting that amylin agonism restores leptin responsiveness in leptin resistance models (85–87). In the long-term, amylin and leptin-co-treated animals exhibited an improved metabolic profile with decreased plasma insulin and plasma lipids, and favorable glucose metabolism (88). Pramlintide acetate, a synthetic analog of amylin and metreleptin, a recombinant methionyl form of human leptin, are analogs of amylin and leptin used in humans. Initial studies suggested that the combined treatment of pramlintide and metreleptin in humans led to a more significant weight loss than treatment with pramlintide or metreleptin alone (89, 90). However, in 2011, Amylin, Inc. and Takeda Pharmaceutical announced in a press release that the development of a pramlintide/metreleptin combination therapy has been stopped due to potential safety concern (see http://www.takeda.com/news/2011/20110805_3889.html).

Other combinations of hormones were recently analyzed to investigate their effect on sensitizing the body to leptin. A recent study proposes a potential role of the gut hormone cholecystokinin (CCK) for the pharmacotherapy of obesity. CCK was shown to synergize amylin-induced suppression of food intake in lean mice (91). The subcutaneous tri-infusion of amylin, leptin, and CCK led to significantly greater weight loss, inhibition of food intake, and reduction in adiposity, compared to the combined treatment with amylin and leptin in DIO rats (92). Apart from amylin and CCK, several studies highlighted the synergistic effect between leptin and the Glucagon like peptide 1 (GLP-1) system in the regulation of energy balance and glucose homeostasis (93–95). In particular, a co-administration of leptin and exendin-4, a natural agonist of GLP-1 receptor, restored leptin responsiveness in DIO mice switched to normal diet, and led to greater body weight loss compared to monotherapy (95). In this study, Muller et al. showed that caloric restriction (even by 30%) does not improve leptin resistance in DIO mice suggesting that weight loss itself is insufficient in restoring leptin-sensitivity and that a combined administration of hormones is needed. Few years later, the same group also reported that coagonism with the GLP-1 and glucagon receptors can restore leptin responsiveness in DIO mice maintained on a HFD (96). Very recently, co-administration of clusterin, a ligand for LDL receptor-related protein-2 (LRP2) potentiated the anorexigenic effect of leptin and boosted leptin-induced hypothalamic STAT3 activation (97). The effects of the co-treatment of leptin with other hormones on energy balance are supposed to arise from the activation of intrinsic synergistic neuronal signaling pathways (86, 98).

Co-administration of leptin along with a hormonal cocktail shows promising results in ameliorating leptin sensitivity. Reversing leptin resistance in DIO conditions with multiple therapies seems now a possible perspective. The latter needs further investigation in order to determine the underlying molecular mechanisms involved in synergistic effects of hormones, as well as the potential beneficial effects in obese humans.

## New Agonists for the Leptin Receptor

The natural hormone leptin is not stable *in vivo* and has a short half-life. Stabilized leptin derivatives or small synthetic OBR agonists would be of great interest in efficiently activating OBR, when the natural ligand becomes inefficient. New OBR agonists could activate the receptor in a similar manner than the natural hormone or even use a different mechanism. Several studies identified numerous leptin-related peptide analogs and mimetics capable of binding and activating OBRb. A synthesized fragment of leptin of 35 amino acids, named OBGRP 22–56 reduced food intake when administrated into the lateral cerebroventricle of rats (99). A series of conjugated molecules built with a peptide vector capable of improving the transport across the blood–brain barrier (BBB), and attached by a linker to a leptin fragment, were patented as new leptin agonists with an improved permeability through BBB. Several generations of such conjugated compounds were synthesized with different linker properties (amino acid sequence or N-substituted succinimide moiety) and different types of leptin sequences (22–56; 57–92; 93–105; 116–130) in order to ameliorate the anti-obesity properties of the molecules (100, 101). Krainov et al. produced other leptin agonists by combining covalently leptin fragments with hydrophilic polymers such as polyethylene glycol (PEG), which increases water solubility and half-life of leptin. Those conjugated peptides have been shown to decrease body weight in mice (102) but alone pegylated leptin is insufficient in improving leptin resistance (95).

Another generation of synthetic leptin peptides referred to as “OB-3” and analogs contains the C-terminal amino acids residues 116–122 (103). Intriguingly, intraperitoneal administration of OB-3 and analogs reduces body weight and food intake in *db/db* mice deficient in OBRb. The authors concluded that the peptides were not acting directly through OBRb and therefore suggested that OB-3 and analogs may have therapeutic applications in clinical situations where administration of recombinant leptin would be inefficient such as in a context of OBR gene mutation. OB-3 and analogs are able to efficiently cross the BBB with an increased bioavailability in the central nervous system (CNS) compared to natural leptin. These synthetic peptides regulate energy balance, but also normalize glycemia and insulin sensitivity in rodent models of obesity (104). The formulation for nasal or oral administration of [D-Leu-4]-OB-3 improves the pharmacokinetical profile compared to intraperitoneal administration, and leads to slight improvement of energy balance and glucose homeostasis (105, 106).

We also put some effort in designing assays searching for small chemical or peptide molecules that would modulate OBR activation. Since no high-resolution crystal structure of the leptin:OBR complex is available to guide the design of such molecules, a screen for positive hits without prior knowledge of the compound/peptide structure is an alternative solution. Two different types of molecules might be identified with such an assay, those potentiating/sensitizing the effect of leptin and those activating OBR by itself in the absence of leptin. In this context, we established a high-throughput screening assay to search for small molecular compounds or peptides and designed a leptin binding assay based on the homogenous time-resolved fluorescence (HTRF) technology (107). HTRF binding assay is based on the energy transfer between a fully functional SNAP-tagged OBR fusion protein (the SNAP protein can be covalently labeled with terbium cryptate, the energy donor) and the biologically active fluorescent leptin-d2 (energy acceptor). The assay is easily miniaturized, robust, sensitive, and non-radioactive. Positive allosteric molecules able to modify or increase leptin binding could hold high potential for helping leptin to activate OBR and reverse leptin resistance. New agonists that would easily cross the BBB in conditions where leptin transport is itself impaired and limited would substitute leptin and efficiently activate neuronal OBR. Recently, Simpson et al. designed pyridine and piperazine derivatives, which showed efficacy in reducing mouse body weight, and proposed that their compounds modulate OBR signaling pathway. However, no robust proof has been provided on the specific target of the compounds (108, 109).

Derivatives of leptin fragments and small molecular compounds targeting OBR could constitute promising tools to improve the activation of OBR, when leptin itself is not active anymore. Such molecules could either better penetrate into the brain than leptin or allosterically activate OBR. Long-term studies would assess whether those molecules can become valuable drugs against leptin resistance and obesity.

## Alleviating the Inhibition of the Leptin Receptor Signaling

Several reports show that negative regulators of leptin signaling, such as the suppressor of cytokine signaling 3 (SOCS3) and the phospho-tyrosine protein phosphatase PTP1B, can be involved in leptin resistance. SOCS3, whose expression is induced by the activation of the JAK2/STAT3 pathway, constitutes a negative retro control mechanism of leptin signaling. SOCS3 binds the tyrosine 985 of OBRb and inhibits leptin-induced phosphorylation of the receptor, an obligatory step of downstream signaling (110, 111). Furthermore, SOCS3 also binds to JAK2 and inhibits its phosphorylation and subsequent activation (54). Thus, a deregulation of SOCS3 expression and activity can disrupt the negative feedback loop in the leptin signaling and be associated with leptin resistance. Indeed, transgenic mice overexpressing SOCS3 in proopiomelanocortin (POMC) neurons showed an impairment of STAT3 signaling in the hypothalamus, associated with increased body weight, fat mass, and food intake (57). In contrast, transgenic mice with OBRb Y985S mutation, which lost SOCS3 binding site, showed decreased food intake, increased leptin sensitivity and are protected from high-fat diet-induced obesity (56).

Concerning PTP1B, this tyrosine phosphatase binds (112) and dephosphorylates JAK2 thus inhibiting downstream leptin signaling both *in vitro* and *in vivo* (55, 113). Ubiquitous, neuron-specific or POMC neuron-specific deletion of PTP1B leads to decreased body weight and fat mass, increased energy expenditure, increased leptin sensitivity, improved glucose homeostasis, and resistance to high-fat diet-induced obesity (114–118). An enhanced PTP1B expression, as seen in the case of ER stress, is associated with leptin and insulin-resistance (58).

Given the involvement of negative regulators of leptin signaling in leptin resistance, decreasing the activity of SOCS3 and PTP1B appears to be a promising way to restore leptin responsiveness in obese people (54, 57, 113–118). Various pharmaceutical companies attempted to develop and optimize PTP1B inhibitors, based on pTyr mimetics (which bind to the active site of PTP1B without being hydrolyzed) or to screen for small molecule inhibitors. Among the small molecular compounds identified, thiazolidinedione derivatives suppressed weight gain and improved lipid-related blood parameters in HFD mice (119). Lantz et al. showed that a treatment with trodusquemine, a selective and allosteric inhibitor of PTP1B, reduced food intake, fat mass, and body weight of DIO mice. This compound crosses the BBB and increases insulin-induced phosphorylation of the insulin receptor and STAT3. Trodusquemine (Genaera Corporation) and PTP1B antisense oligonucleotides (ISIS Pharmaceuticals) are currently in phase II clinical trials as a potential treatment for obesity and diabetes (58, 120). To our knowledge, specific SOCS3 inhibitors do not exist. As a future perspective, we propose a strategy based on the prevention/disruption of the interaction between OBR and SOCS3. Preventing the interaction between SOCS3/OBRb is supposed to alleviate the inhibition of JAK2 and OBRb phosphorylation, and thus prolong and increase the activation of the leptin signaling cascade. Targeting the interaction between OBR and SOCS3 should be a more specific means to achieve SOCS3 inhibition at the level of the OBR pathway. The discovery of such inhibiting molecules (chemical compounds or peptides) can be achieved via screening of compounds that would interfere with the interaction between OBRb and SOCS3. Bioluminescence resonance energy transfer (BRET) has been widely used to study protein–protein interaction (121–123) and to screen for drugs that would increase or decrease protein interactions (124, 125). BRET-based assay for high-throughput screening could be foreseen as a promising strategy to look for peptide or compounds that would interfere with OBR and SOCS3 interaction, and by extension with OBR and PTP1B.

## Increasing the Pool of Surface Receptors Accessible to Leptin

Several reports suggested that OBR is mainly found in the cytoplasm rather than at the cell surface of hypothalamic neurons (126–128). Studies in transfected cells have confirmed that OBR localization is predominantly intracellular (129, 130). Surface localized OBR is constitutively internalized in a ligand-independent manner and clathrin-mediated endocytosis (131, 132). The constitutive internalization of OBR involves several signals located in the cytoplasmic tail of OBR, in particular two lysine residues in OBRa have been identified that are subject of ubiquitination and required for constitutive receptor endocytosis. The study of Belouzard et al. described more precisely the intracellular distribution and trafficking of OBRa and OBRb, which follow similar intracellular routes. After internalization, OBR is found in a peripheral compartment corresponding to EEA1 and Rab5-positive early endosomes, before being targeted toward lysosomal degradation (131). Some neosynthesized receptors are retained in the Trans-Golgi network (TGN), through signals contained in the transmembrane-domain of the receptor, before being targeted toward lysosomes. The constitutive internalization, the intracellular retention in the biosynthetic pathway and the low recycling rate of OBR result in low levels of receptor expression at the cell surface, which is critical for leptin-sensitivity. Indeed, only 5–25% of OBR are expressed at the plasma membrane (129). In this regards, defects in leptin receptor trafficking could alter proper leptin receptor signaling (40, 133, 134).

The interaction of OBR with trafficking proteins regulates its intracellular trafficking, and consequently its cell surface expression and ability to bind leptin. This constitutes an important issue since the pool of OBR at the cell surface determines the sensitivity of the cell to leptin. OBRb, but not the short OBR isoforms, was previously shown to interact with the sorting molecules, nexin1, nexin4, and nexin6 (135, 136); however, the importance of those interactions has not yet been assessed. A recent study identified that by interacting with OBR and the deubiquitinase USP8 (necessary for stabilizing the ESCRT-0 complex components), the ubiquitin ligase RNF41 controls OBR trafficking among other cytokine receptors. RNF41 leads to an increase of OBR recycling to the plasma membrane versus lysosomal targeting (137). Conversely, in cultured neurons, clusterin, and LRP2 enhance OBR and leptin endocytosis, necessary for hypothalamic STAT3 activation (97). We also previously identified endospanin 1, a protein encoded by the *db* gene by alternative splicing. Endospanin 1, by directly interacting with OBR specifically, regulates OBR cell surface expression by retaining a significant amount of OBR in intracellular compartments (133, 138). Even a twofold increase in OBR surface expression with endospanin 1 knock-down was sufficient to significantly increase leptin-induced STAT3 phosphorylation *in vitro* and *in vivo* in the ARC of the hypothalamus, and was efficient in preventing or reversing the development of obesity in mice fed with a high-fat diet (133, 139). This observation suggests that mobilizing OBRs from intracellular pools can modulate leptin-sensitivity in neurons. This proof of concept leads to a new therapeutic strategy to restore leptin-sensitivity in obese patients. Given the efficacy of increasing OBR cell surface expression in the ARC neurons, we performed a high-throughput phenotypic imaging-based screening by confocal microscopy and identified small molecular compounds capable of increasing OBR cell surface expression (140). The precise action mechanism of these hits remains to be determined but includes most likely cellular processes that modify OBR redistribution, like endospanin 1 or RNF41 or others, without affecting OBR neosynthesis or degradation since treatment with these compounds did not modify the net expression of total OBR. The positive hits might play a role in decreasing OBR internalization, increasing OBR recycling, and enhancing OBR transport to the cell surface. Importantly, the compounds identified increase the signaling capacity of leptin by enhancing leptin-induced activation of the STAT3 pathway. The therapeutic potential of these compounds has now to be determined in the context of leptin resistance and obesity treatment.

## Increase Leptin Transport through the Blood–Brain Barrier

The BBB, formed at the level of brain microvessel endothelium with tight junctions, is the major site of exchange between the periphery and the CNS, while the barrier between the blood and cerebrospinal fluid (CSF) lies at the choroid plexuses in the lateral, third, and fourth ventricles of the brain. To reach OBRs in the brain, leptin must be transported across the brain barrier most likely *via* a specific, saturable, and unidirectional transport system (141). This system is supposed to be located at the endothelium of cerebral microvessels and/or the epithelium of the choroid plexus (142, 143). The choroid plexus, responsible for the production of the CSF, is also involved in the transport of many peptide hormones such as insulin (144). Leptin release from the choroid plexus into the CSF was supposed to be mediated by transcytosis of a transporter. A myriad of evidence suggests that the highly expressed OBRa and OBRc isoforms are involved in the transport of leptin across the BBB or choroid plexus (12, 142, 145–147). A recent study identified the potential leptin transporter at the choroid plexus epithelium, as being LRP2 (also known as megalin), which captures circulating leptin and transports the hormone into the brain (148).

Since a long time, several reports have suggested that leptin resistance was associated with a defect in the transport of leptin through the BBB. DIO mice have the ability to respond to centrally administrated leptin, by reducing food intake, but they do not respond to a peripheral administration of exogenous hormone (47). Banks et al. showed that the transport rate of leptin through the BBB is diminished by about 2/3 in obese CD-1 mice (48). Reduction of leptin uptake by the brain of New Zealand Obese mice, a strain that responds to central but not peripheral leptin, suggested that their obesity is at least partly due to deficient leptin transport into the brain (142). These studies strongly suggest that a defective transport of leptin across the BBB is correlated with leptin resistance and obesity. The defect in leptin transport was also observed in obese patients, in which the CSF/serum leptin ratio is decreased by fourfold compared to lean patients (45, 46). Several hypotheses were proposed for the mechanism(s) of this defective transport of leptin. In 2005, Oh et al. suggested that polyunsaturated fatty acids induce peripheral leptin resistance via an increase in the expression of hypothalamic occludin, one of the main proteins of the tight junctions, reducing paracellular transport of leptin into the brain (149). An impaired expression of the transporters OBRa and OBRc at the BBB and the choroid plexus was also suggested but this aspect is quite controversial. Some studies revealed that high-fat diet or leptin challenge had no effect on OBR mRNA levels in the choroid plexus (150) neither in cerebral microvessels of mice (142). On the contrary, Boado et al. observed a strong increase (11-fold) of the mRNA levels of OBRa (not OBRb) in brain capillaries of HFD rats, an observation that was confirmed at the protein levels by immunohistochemistry. However, in this study, the serum leptin levels of HFD rats were similar to those in the control group (151). Finally, several studies suggested that the defect of leptin transport in obese mice is due to a decreased capacity of the transporter to bind and transport leptin into the brain, and not only a saturation of the transporter by hyperleptinemia (48, 150, 152). More precisely, this hypothesis is nicely illustrated by the study of Banks and colleagues where they observed that, by replacing the hyperleptinemic blood of obese mice with a buffer containing low concentrations of radiolabeled leptin, obese mice had still lower transport rate of leptin through the BBB than lean mice (48).

Recently, a new path for leptin transport into the hypothalamus was discovered. It is believed that circumventricular organs, such as the *median eminence* (ME), which lack a typical BBB, allow free diffusion of molecules and peptides through their fenestrated capillaries. In particular, the OBR-expressing neurons of the ARC are in very close proximity to these fenestrated capillaries at the basis of the hypothalamus (153). Tanycytes are specialized hypothalamic glial cells located in the ME and extending from the ependymal surface of the third ventricle to fenestrated vessels at the pial surface of the brain. Remarkably, at the level of the ME, plasma leptin is first uptaken by the feet of tanycytes, traffics to the apical end, before being released in the CSF, and targeting neurons of the mesobasal hypothalamus (154). Interestingly, leptin release in the CSF is dependent on the activation of ERK signaling, a pathway impaired in DIO mice. Restoring ERK signaling and leptin release in the CSF, is able to recover leptin-induced hypothalamic STAT3 activation and accelerate weight loss in leptin-resistant DIO mice switched to chow diet (154).

Valuable tools to increase leptin entry into the brain would be strategies that would selectively improve the leptin transport across the BBB without disturbing the overall intactness of the barrier. Strategies favoring leptin passage into the brain include the design of leptin analogs or new OBR agonists with improved BBB permeability (155). Several strategies have been used to improve circulating half-life, potency, solubility, and permeability of leptin. Modified leptin with PEG, even with an increased half-life, is unable to cross the BBB (156) and has no effect on body weight of obese human (157). However, other modifications of leptin were shown relevant as therapeutic strategies for obesity: indeed, leptin carrying a carbohydrate moiety (158) and leptin modified with trans-activating transcriptional activator (TAT) (159) or with Pluronic^®^ (155, 160) have increased transport into the brain and weight loss in DIO mice. Some effort still needs to be provided in this sense. In the light of the ERK-dependent leptin release into the third ventricle by the ME tanycytes, any pharmacological treatment that would reactivate ERK signaling in impaired tanycytes would hold promising therapeutic potential against leptin resistance sustained by a diminished central leptin transport. Collectively, until recently, the mechanisms by which the brain takes up leptin remained unclear. Recent studies brought new light on the molecular and cellular nature of the transporting system, underlying the importance of LRP2 and tanycytes. Such discoveries warrant further investigation in humans.

## Decreasing ER Stress with Chemical Chaperones

The ER is an important organelle regulating the synthesis, folding, maturation, quality control, and traffic of proteins. Alteration of proper ER function triggers a state of “ER stress” and leads to activation of the “unfolded protein response (UPR)”, in order to adapt the secretory apparatus and cellular physiology in response to stress (161). The UPR signaling has a protective role by (i) augmenting the expression of proteins involved in the folding machinery, (ii) increasing the rate of protein folding, and (iii) diminishing the overall translation of proteins for attenuation of ER load. Misfolded proteins are then targeted for degradation by the proteasome. However, persistent and prolonged UPR signaling would trigger cell death. UPR signaling is mediated by three main ER transmembrane proteins, IRE1 (inositol-requiring protein-1), PERK (protein kinase RNA-like ER kinase), and ATF6 (activating transcription factor-6).

Persistent ER stress and UPR signaling have been implicated in various diseases including metabolic disorders such as obesity and type 2 diabetes (162). Cholesterol and free fatty acids were shown to induce ER stress (163–165). ATF6 seems to be involve in the regulation of glucose metabolism in the liver and pancreas (166, 167). PERK pathway has an important role in pancreatic β cell function since PERK-null mice or mice with pancreas-specific deletion of PERK develop hyperglycemia with the loss of pancreatic islets (168–170). Recent studies demonstrated that ER stress is involved in the development of leptin resistance by abrogating leptin signaling (64, 65). Pharmacologically induced ER stress suppresses leptin signaling in the hypothalamus and increases food intake and body weight in mice (65). Neuronal deletion of XBP1, a IRE1-regulated transcription factor, triggers hypothalamic ER stress, and leptin resistance associated with weight gain upon high-fat diet (65). ER stress in the brain also contributes to central insulin-resistance along with leptin resistance (66).

Chemical chaperones constitute a promising therapeutic treatment against leptin resistance in order to re-sensitize the cells to leptin. They are small molecular compounds that stabilize the folding of proteins and help to decrease abnormal protein aggregation and reduce ER stress. 4-phenylbutyrate (4-PBA) and tauroursodeoxycholic acid (TUDCA) are the best-known chemical chaperones with FDA approval. 4-PBA and TUDCA treatment successfully alleviate hypothalamic ER stress and restore leptin responsiveness in high-fat diet-fed mice by reducing food consumption along with body weight (65). Hosoi et al. has reported that fluvoxamine, a selective serotonin reuptake inhibitor prescribed for the treatment of depression, which binds the ER receptor Sigma1, is able to alleviate ER stress and reverse leptin resistance in cells, and to decrease food intake in mice (171). Recently, flurbiprofen, a non-steroidal anti-inflammatory drug (NSAID), was shown to reduce ER stress, to attenuate leptin resistance and to reduce the body weight of HFD obese mice (172, 173). The authors suggested that flurbiprofen reduces the aggregation of aldehyde dehydrogenase, leading to the reduction of ER stress and of leptin resistance (172).

Promisingly, chemical chaperones gave encouraging results when administrated for the treatment of human obesity and diabetes. 4-PBA and TUDCA were demonstrated to improve insulin signaling and glucose homeostasis in human obese subjects characterized by insulin-resistance, and improved insulin sensitivity in obese subjects (174, 175). Those observations in humans suggest that alleviating ER stress constitutes a potential therapeutic strategy for metabolic diseases like obesity and type 2 diabetes.

## For Other Type of Disorders

Leptin exerts also direct effects on peripheral organs and regulates a wide range of functions including immunity (176, 177), neuroendocrine functions (178), bone formation (179), reproduction (180), and angiogenesis (181, 182). Given the involvement of leptin in such diverse biological effects, leptin resistance and obesity are frequently associated with other diseases such as autoimmunity, some cancers, cardiovascular, and neurodegenerative diseases. Indeed, several evidence suggest a neuroprotective role of leptin, which can prevent neuronal death in various neurodegenerative situations, such as models of Parkinson’s disease (183, 184), Alzheimer’s disease (AD) (185), and models related to epilepsy (186). Leptin can also modulate synaptic plasticity involved in memory formation: mouse models of AD treated with exogenous leptin show improved memory (187). A recent study emphasizes an active role of leptin signaling in astrocytes for the regulation of synaptic plasticity and feeding (188). Furthermore, glial cells play a central role in protection from neuro-inflammation, process involved in neurodegenerative situations (189). In this context, new agonists for OBR or OBR sensitizing molecules would be promising therapeutic tools for neurodegenerative diseases. On the other hand, because leptin promotes autoimmunity (190, 191), cancer cell growth and migration, the identification of new OBR antagonists would be of interest in the field of autoimmune disease and cancer therapy (192–194).

In this review, we attempted a summary of recent pharmacological tools developed for OBR. These efforts will continue at the speed at which novel facets of OBR function will be discovered. For instance, new discoveries keep underlying the importance of interacting proteins or lipids for OBR signaling. For example, increased association of gangliosides with OBR upon leptin stimulation participates in receptor activation (195). Furthermore, the low-density lipoprotein receptor-related protein-1 (LRP1) was demonstrated to bind to the leptin:OBR complex and suggested to be necessary for OBR phosphorylation and STAT3 activation (196). Similarly, clusterin and LRP2 are critical for leptin endocytosis and leptin-induced STAT3 activation in the hypothalamus (97). Endospanin 1 and RNF41 regulators control OBR trafficking and signaling. Almost all those interacting molecules have been shown to be implicated in the leptin regulation of body weight. They could be included in the effort of finding leptin sensitizing mechanisms/molecules. Important efforts have been made to identify the origin of leptin resistance and mechanisms that sustain it, such as hyperleptinemia, failure of central transport of leptin, or ER stress. Therapeutic tools or concepts have also been developed, either with low or strong efficacy, in order to ameliorate leptin-sensitivity and reverse its loss. In the long-term, discovery of new drugs against leptin resistance, acting at multiple levels, should be included in a therapeutic setting where combinatorial treatment in association with other hormonal therapies should help decreasing food intake and increasing energy expenditure and therefore leading to steady reversal of obesity with limited risks.

## Conflict of Interest Statement

The authors declare that the research was conducted in the absence of any commercial or financial relationships that could be construed as a potential conflict of interest.
